# Trans-Graft Protection Against Pierce’s Disease Mediated by Transgenic Grapevine Rootstocks

**DOI:** 10.3389/fpls.2019.00084

**Published:** 2019-02-06

**Authors:** Abhaya M. Dandekar, Aaron Jacobson, Ana M. Ibáñez, Hossein Gouran, David L. Dolan, Cecilia B. Agüero, Sandie L. Uratsu, Robert Just, Paulo A. Zaini

**Affiliations:** ^1^Department of Plant Sciences, University of California, Davis, Davis, CA, United States; ^2^Department of Enology and Viticulture, University of California, Davis, Davis, CA, United States

**Keywords:** grapevine, transgenic rootstock, Pierce’s disease, trans-graft protection, *Xylella fastidiosa*

## Abstract

A field study showed that transgenic grapevine rootstocks can provide trans-graft-mediated protection to a wild type scion against Pierce’s disease (PD) development. We individually field-tested two distinct strategies. The first expressed a chimeric antimicrobial protein (CAP) that targeted the functionality of the lipopolysaccharide (LPS) surface of *Xylella fastidiosa* (*Xf*), the causative agent of PD. The second expressed a plant polygalacturonase inhibitory protein (PGIP) that prevents PD by inhibiting breakdown of pectin present in primary cell walls. Both proteins are secreted to the apoplast and then into the xylem, where they migrate past the graft union, transiting into the xylem of the grafted scion. Transgenic *Vitis vinifera* cv. Thompson Seedless (TS) expressing ether CAP or PGIP were tested in the greenhouse and those lines that showed resistance to PD were grafted with wild type TS scions. Grafted grapevines were introduced into the field and tested over 7 years. Here we present data on the field evaluation of trans-graft protection using four CAP and four PGIP independent rootstock lines, compared to an untransformed rootstock. There was 30 to 95% reduction in vine mortality among CAP- and PGIP-expressing lines after three successive yearly infections with virulent *Xf*. Shoot tissues grafted to either CAP or PGIP transgenic rootstocks supported lower pathogen titers and showed fewer disease symptoms. Grafted plants on transgenic rootstocks also had more spring bud break following infection, more shoots, and more vigorous growth compared to those grafted to wild type rootstocks. No yield penalty was observed in the transgenic lines and some PGIP-expressing vines had enhanced yield potential. Trans-graft protection is an efficient way to protect grape scions against PD while preserving their valuable varietal genotypes and clonal properties.

## Introduction

Starting in the 1850s, an epidemic of *Phylloxera* swept through the vineyards of Europe, crippling its wine industry. The solution to this problem was to graft *Vitis vinifera* scion cultivars to *Phylloxera*-resistant rootstocks. Without grafting and the identification of resistant rootstocks, there would be no *V. vinifera*-based wine industry in Europe today. Worldwide, vineyards are planted almost exclusively with composite vines: locally adapted rootstocks that provide disease and pest resistance are grafted with grapevine scion varieties that produce high-quality varietal wines. We have expanded the concept of rootstock-mediated resistance to include engineered disease resistance (Agüero et al., 2005; Dandekar et al., 2012). This strategy is particularly useful for diseases that target roots, such as bacterial crown gall disease (Escobar et al., 2001; Escobar and Dandekar, 2003).

Pierce’s disease (PD) of grapevines is an insect-transmitted bacterial disease caused by *Xylella fastidiosa* (*Xf*). It is one of the most serious aboveground grapevine diseases and is the primary reason why it is difficult to grow grapevines in the southeastern United States. Infected vines develop scorched leaves and shriveled berries, with plant death occurring within a few years (Hopkins and Purcell, 2002). Xylem sap-feeding sharpshooter insects are the vectors that transmit PD during feeding. Once in the xylem, the pathogen uses surface adaptations such as pili and other adhesive molecules to move freely with or against the flow of xylem sap (Meng et al., 2005; Chatterjee et al., 2008a; Clifford et al., 2013). The prevailing hypothesis for disease development is water deficit, caused by blockage of the xylem elements by bacterial biofilms, combined with host responses such as tyloses and pectin gels (Purcell and Hopkins, 1996; Chatterjee et al., 2008a). This hypothesis fails to capture the true complexity of this host-microbe interaction, as it relies solely on physical aspects but neglects chemical reactions occurring during infection and host colonization. It doesn’t explain for example why biofilm-deficient *Xf* mutants display hypervirulence (Guilhabert and Kirkpatrick, 2005; Chatterjee et al., 2008b; Gouran et al., 2016). The xylem occlusion hypothesis also doesn’t explain the battery of enzymes and other compounds secreted by the pathogen that modify the xylem microenvironment and trigger chronic host stress responses (Roper et al., 2007; Pérez-Donoso et al., 2010; Pierce et al., 2014; Gouran et al., 2016; Merfa et al., 2016; Nascimento et al., 2016; Assis et al., 2017; Labroussaa et al., 2017; Zaini et al., 2018). These studies are broadening our understanding of virulence strategies employed by *Xf* and of how it thrives in the nutrient-poor xylem environment. Among the enzymes secreted by *Xf* are glycan-degrading enzymes that modify an array of host, vector, and microbial moieties, occurring on the interfaces of pathogen and host xylem, or insect foregut. They occur as *N*-glycans decorating host proteins involved in pathogen perception (Häweker et al., 2010) and also on the structural polymer pectin (Harholt et al., 2010). Plant cells can sense and trigger responses to damage-associated molecular patterns. If sustained, this can lead to chronic oxidative stress and cell death consistent with the leaf scorching symptoms observed with PD.

Many of the commercial *V. vinifera* varieties are highly susceptible to PD. The current disease control strategy is intense management with chemical pesticides to target the insect vector, but not the bacterium that causes the disease. As current insecticides gradually lose efficacy, novel compounds and/or control methods are needed (Andreason et al., 2018). Although there has been progress in biocontrol strategies (Zhang et al., 2015), genetic resistance, as found in natural populations of other *Vitis* species such as *V. arizonica* and *V. rotundifolia* (muscadine), could protect *V. vinifera* varieties with high efficiency and low maintenance. While breeding in resistance genes from tolerant species remains promising, an alternative approach is to engineer resistance with trans or synthetic genes (Agüero et al., 2005; Dandekar et al., 2012; Lindow et al., 2014). Transgenic grapevines expressing a pear polygalacturonase inhibitory protein (PGIP) (Agüero et al., 2005) or a chimeric antimicrobial protein (CAP) (Dandekar et al., 2012) were tolerant of PD. These recombinant proteins were targeted into the xylem lumen, in which they traverse the graft union and are detectable in sap extracted from the scion (Agüero et al., 2005; Dandekar et al., 2012). Most proteins secreted to the apoplast travel though the endomembrane system, the primary route outside the plant cell (Drakakaki and Dandekar, 2013). The presence of a signal peptide is a key feature of proteins found in grapevine xylem (Agüero et al., 2008). Here we explore this targeting mechanism and investigate the resistance to PD of multiple lines expressing different PGIP and CAP constructs in field trials.

## Materials and Methods

### Transgenes, Binary Vectors and Grapevine Transformation

The construction of the CAP-1 transgene expressing vectors pDU04.6105 and pDA05.0525 was described previously (Dandekar et al., 2012). The binary vector pDU94.0928, which expresses pear PGIP using its endogenous signal peptide (Stotz et al., 1993), was described previously (Agüero et al., 2005). A pear PGIP transgene without its endogenous signal peptide, mature polygalacturonase inhibiting protein (mPGIP), was created by amplifying the coding sequence from pDU94.0928 using the primers: 5′ mPGIP: 5′ ATGGATCTCTGCAACCCCGACGAC and 3′PGIP: 5′ TTACTTGCAGCTTGGGAGTG and cloning the PCR products into the pCR2.1-TOPO vector (Invitrogen, Carlsbad, CA, United States). The mPGIP sequence was excised from this vector using restriction endonuclease cleavage and ligated into pDU99.2215 (Escobar et al., 2001), downstream from a CaMV35S promoter and upstream from a ocs3′ regulatory sequence, to create pDU05.1002, or PGIP-2 (Supplementary Figure 1). The signal peptide sequence CHI and NT, corresponding to the Chi1b and NtPRp27-like grapevine xylem-secreted proteins, was amplified from grapevine genomic DNA. The protein sequence was matched in the TGI *Vitis vinifera* gene index^1^ to obtain a DNA sequence, which was used to design primers to amplify the predicted fragments from genomic DNA of “Chardonnay” and “Cabernet Sauvignon” (Agüero et al., 2008). The signal peptide cut site was determined as described (Nielsen et al., 1997). The amplified DNA fragments contained the sequence of each signal peptide, fused in frame to the DNA sequences that contained the mPGIP coding sequence using PCR-based overlap extension (Horton et al., 1990). The PCR products were cloned into the pCR2.1-TOPO vector as described above and then cloned into pDU99.2215 to create the binary vectors pDU05.1910 (PGIP-3) and pDU06.0201 (PGIP-4), which fused the signal peptide sequences for NtPRp27-like and Chi 1b in-frame with mPGIP, respectively. A similar approach was used to incorporate the grapevine version of a signal peptide for a common xylem secreted protein, XSP (Buhtz et al., 2004). The binary vector pDA05.XSP was created and designated PGIP-5 (Supplementary Figure 1). We also incorporated the signal peptide RA3D for the rice alpha-amylase protein from rice designated RAmy3D. We PCR-amplified DNA corresponding to a previously described signal peptide sequence from rice cultures (Trexler et al., 2002), using a method similar to that for the other signal sequences. The binary vector pDU05.0401 was created and designated PGIP-6 (Supplementary Figure 1). All of the binary vectors were transformed into the disarmed *A. tumefaciens* strain EHA 105 pCH32 (Hamilton, 1997) by electroporation (Shen and Forde, 1989) and stable transformation of *Vitis vinifera* “Thompson Seedless” (TS) was performed at the UC Davis Plant Transformation Facility using our *Agrobacterium*-mediated grapevine transformation protocol (Agüero et al., 2006). A molecular and biochemical analysis was performed on each transgenic grapevine plant representing individual transgenic events selected for each binary vector as described (Agüero et al., 2005, 2006 and Dandekar et al., 2012). The presence/absence of each transgene was identified using PCR amplification of the entire coding regions of the genes encoding resistance to kanamycin (Kan), expression of β-glucuronidase (GUS), PGIP, and NE-CecB genes in DNA isolated from each transformation event. Biochemical analysis was performed to confirm GUS (Jefferson, 1987) and PGIP activity (Taylor and Secor, 1988).

### Propagation and Field Planting of Grapevines

Transgenic TS grapevine plants were confirmed by molecular and biochemical analysis and transferred to the greenhouse after acclimatization. In the greenhouse, each individual transgenic event was propagated vegetatively to create plant materials for the field. Each transgenic line was maintained as two mother plants that provided source materials for both transgenic grapevines and transgenic rootstocks to which a wild type scion was grafted. Stem cuttings with at least two nodes were harvested from the mother plants, induced to root in a mist bed, and then transferred to one gallon pots to create transgenic grapevine plants. Wild type TS grapevine stem cuttings of the same diameter were harvested from greenhouse-grown mother plants and bench-grafted to the transgenic rootstocks. These plants were grown in the greenhouse and overwintered in a lath house prior to field planting.

Ninety-six transgenic grapevine rootstocks with grafted wild type scions, corresponding to eight independent transgenic events (Table 1), and 16 controls were planted in Field 1. These 112 vines were planted in five rows of 25 vines each and spaced 2.44 m (8 ft) apart within the rows with 3.1 m (10 ft) between rows. A map of the Field 1 planting is provided (Supplementary Table 1). Forty-eight vines were CAP-1 lines, consisting of 12 replicates each from four lines: CAP-1.041, CAP-1.089, CAP-1.092, and CAP-1.151 (Table 1). Another 48 vines consisted of 12 replicates each of four PGIP events: PGIP-1.50, PGIP-2.25, PGIP-3.08, and PGIP-4.77 (Table 1). The remaining 16 vines were wild type TS rootstocks with grafted wild type TS scions. Each event introduced into Field 1 was represented by 12 biological replicate vines planted in a randomized plot design, with each line represented as four rows of three vines each in a row (Plot map; Supplementary Table 1). A second field planting at a location ∼ 1.5 miles away from Field 1 contained 599 vines planted in 10 rows of 62 plants per row, with 0.9 m (3 ft) between vines and 2.44 m (8 ft) between rows (Table 2). This field planting contained 42 CAP-1 lines, one PGIP-1, eight PGIP-2, six PGIP-3, eight PGIP-4, five PGIP-5, and seven PGIP-6 lines. All lines were planted on their own roots, with six replicate vines for each event planted in two rows of three vines each in a random plot design (Supplementary Tables 2 and 3). The plot had 140 wild type TS vines planted in two rows along two borders.

**Table 1:** Genotypes of lines planted in Field 1.

No	Line designation	Event No.	No of vines	Transgene	Binary vector	Reference
1	CAP-1.041	0999040-41	12	HNE-CecB	pDU04.6105	Dandekar et al. (2012)
2	CAP-1.089	0999040-89	12	HNE-CecB	pDU04.6105	Dandekar et al. (2012)
3	CAP-1.092	0999040-92	12	HNE-CecB	pDU04.6105	Dandekar et al. (2012)
4	CAP-1.151	0999041-151	12	HNE-CecB	pDU04.6105	Dandekar et al. (2012)
5	PGIP-1.50	TS-50	12	pearPGIP	pDU94.0928	Agüero et al. (2005)
6	PGIP-2.25	061131-25	12	mPGIP	pDU05.1002	This Study
7	PGIP-3.08	061052-08	12	ntPGIP	pDU05.1910	This Study
8	PGIP-4.77	061045-77	12	chiPGIP	pDU06.0201	This Study
9	TS-WT		16			
	Total vines planted		112			

**Table 2:** Genotypes of lines planted in Field 2.

No	Line designation	No. of lines	No of vines	Transgene	Binary vector	Reference
1	CAP-1	34	204	HNE-CecB	pDU04.6105	Dandekar et al. (2012)
2	CAP-1	8	48	pHNE-CecB	pDA05.0525	Dandekar et al. (2012)
3	PGIP-1	1	6	pearPGIP	pDU94.0928	Agüero et al. (2005)
4	PGIP-2	8	48	mPGIP	pDU05.1002	This Study
5	PGIP-3	6	36	ntPGIP	pDU05.1910	This Study
6	PGIP-4	8	48	chiPGIP	pDU06.0201	This Study
7	PGIP-5	5	30	xspPGIP	pDA05.XSP	This Study
8	PGIP-6	7	39	RamyPGIP	pDU05.0401	This Study
9	TS-WT		140			
	Total vines planted		599			

### Evaluation of Varietal Characteristics

Individual vines planted in Fields 1 and 2 were evaluated in each of the first three years for 12 descriptors from the “Primary descriptor priority list” proposed by the *Organization Internationale de la Vigne et du Vin* (OIV, 1983). The 12 descriptors evaluated were: (1) aperture of young shoot tip/opening of young shoot tip, (2) density of prostrate hairs between main veins on fourth leaf lower side of blade, (3) number of consecutive shoot tendrils, (4) color of upper side of blade on fourth young leaf, (5) shape of mature leaf blades, (6) number of lobes on mature leaf, (7) area of anthocyanin coloration on main veins on upper side of mature leaf blades, (8) shape of teeth on mature leaves, (9) degree of opening of mature leaves/overlapping of petiole sinuses, (10) mature leaf petiole sinus bases limited by veins, (11) density of prostrate hairs between main veins on lower side of mature leaf blades, and (12) density of erect hairs on main veins on lower sides of mature leaf.

### Inoculation of Vines, Titer Estimation and Disease Phenotyping

Temecula 1 and Stag’s Leap strains of *Xf* were used for all vine inoculations in Field 1. The bacteria were grown on PW medium (Davis et al., 1981) and resuspended to 1.25 to 2.5 × 10^7^ cells/mL as described (Dandekar et al., 2012). Individual shoots were inoculated 10 cm above the cordon by piercing an 18-gauge needle through the center of the shoot, depositing 20 μL inoculum culture on the tip of the needle that emerged on the other side, then drawing the needle back to allow the inoculum to be sucked into xylem. After being planted in the field all of the vines were grown without inoculating. In year 2, 6 of the 12 vines for each line were inoculated, in year 3 the remaining 6 were inoculated and in years 4 and 5 all vines were inoculated. Inoculations were performed yearly in late spring in the second through fifth years. A 60:40 mixture of Temecula 1 and Stags Leap strains was used in the second and fourth years and Temecula 1 was used in the third and fifth years. Bacterial titer was estimated by qPCR in a one-inch stem segment 10 cm above the point of infection. Stem samples were harvested from infected vines 2 months and 1 year after infection and ground into a powder in liquid nitrogen. DNA was extracted using a modified CTAB (hexadecyltrimethyl-ammonium-bromide) method (Lodhi et al., 1994). The *Xf* 16s primer pair (forward 5′-AATAAATCATAAAAAAATCGCCAACATAAACCCA-3′ and (reverse 5′- AATAAATCATAACCAGGCGTCCTCACAAGTTAC-3′) was used for *Xf* quantification. qPCR standard curves were obtained using concentrations of *Xf* ranging from 10^2^ to 10^6^ cells per 0.1 g tissue. After converting CT values to CFUs using a generated standard curve, values greater than 10 CFU were considered positive for infection and used to analyze the titer. Samples where CFU was less than 10 were not considered as 10 was determined to be the minimum reliable detection limit. Vine health was assessed in spring by evaluating bud break. The inoculated canes that were not removed to measure bacterial titer were allowed to overwinter and were not removed from the grapevines during winter pruning. These canes were monitored in the spring as plants began to break dormancy. As xylem sap began to flow into canes and buds began to swell, they were observed for differences between transgenic and control vines. As buds swelled and began to break open, many buds on infected canes either failed to break or dried up and aborted. Once buds developed past three or four leaves, they usually survived. At this time, the proportion of successfully emerged and developing buds on each cane was determined. The percentage bud break was calculated for each shoot by counting the number of buds that swelled, broke open, and did not abort as a percentage of the total number of buds on each cane on each plant (*n* = WT-TS: 27, CAP1.041: 22, CAP1.089: 20, CAP1.092: 19, CAP1.151: 31, PGIP-1.50: 27, PGIP-2.25: 24, PGIP-3.08: 29, PGIP-4.77: 25). Vine mortality was assessed in the fall by observing each grapevine for any sign of survival. A vine with any amount of live tissue was counted as surviving. A deceased plant was completely defoliated and dried up. Mortality was calculated as a ratio of living/dead plants, with *n* = 12 for each line.

### Evaluation of Horticultural Traits

The yield from each grapevine planted in Field 2 was evaluated in the sixth and seventh years. Total kg fruit from each vine was harvested into a basket and weighed in the field using a mobile scale. Statistical significance of the differences observed between transgenic and wild type controls was calculated using an unpaired *t* test.

### Radial Diffusion Assay

Xylem sap was collected from 6 vines corresponding to each of the 33 PGIP-expressing grape lines and from 6 WT lines in Field 2. In the spring of year 7 shoots at the top of the vines were cut at the tip and 30 to 50 ml of xylem sap was collected in a 50 ml Falcon tube. The xylem sap was centrifuged at 15,000 xg for 10 min at 4°C and then aliquoted and frozen at −80°C until analysis. PG preparation was obtained from *Botrytis cinerea* strain Del 11 isolated from grapevine as described (Agüero et al., 2005). The xylem sap samples were assayed for percentage inhibition of *Botrytis cinerea* PG activity by a semiquantitative radial diffusion assay in agarose (Taylor and Secor, 1988). Each assay medium plate contained 5 mg potassium pectate (Sigma) as the substrate, 0.5 g SeaKem LE agarose (Lonza Rockland, Inc., Rockland, ME, United States), and 372 mg EDTA (Sigma-Aldrich) in 50 mL 0.1 M sodium acetate buffer at pH 5. The medium was microwaved to dissolve the potassium pectate and agarose, then transferred to 3.5″ square petri polystyrene plate (50 mL per plate). The medium solidified at room temperature and a 5 mm cork borer was used to punch wells in which the PG enzyme-PGIP inhibitor reaction took place. The inhibition of PG by PGIP was measured in a 40 uL mixture of PG and xylem sap from PGIP-expressing lines per well, using PG and TS-WT as positive and negative controls, respectively. The assay was incubated at 37°C for 18 h, then enzymes were denatured with 0.5 N HCl and the clear zone of PG activity was developed by flooding the assay plate with 25 mL of 0.05% ruthenium red (Sigma-Aldrich) for 30 min at room temperature. Assay plates were washed several times with deionized water to remove excess dye until the clear activity zone was developed. Seven technical replicates per sample were performed and the resulting clear ring of PG activity was measured and averaged, then compared to the PG control to calculate the percentage reduction of the radius of the clear zone as a measure of PGIP efficacy.

## Results

This study builds on two earlier studies which demonstrated that grapevines expressing either a CAP (Dandekar et al., 2012) or a PGIP showed tolerance to PD (Agüero et al., 2005). In both studies, CAP and PGIP proteins were secreted to the xylem, where they provided protection against PD. This study examines whether these plants when used as rootstocks can provide trans-graft protection to a wild type, PD-sensitive scion under field conditions.

### Evaluation of Varietal Characteristics of Field Grown Transgenic Grapevines Expressing CAP of PGIP Proteins

One hundred and twelve grafted grapevines in Field 1 were evaluated for their response to PD. Fourty eight of these corresponded to four CAP lines (CAP-1.041, CAP-1.089, CAP-1.092, and CAP-1.151), 48 corresponded to four PGIP and 16 vines were TS wild type rootstock grafted to TS wild type scion to serve as controls. The other four PGIP-expressing lines (Table 1) included PGIP proteins with different signal peptides (see Methods for complete description). Field 2 contained 599 vines and included 42 lines of CAP-1 and 35 lines of PGIP (Table 2).

**FIGURE 1:**
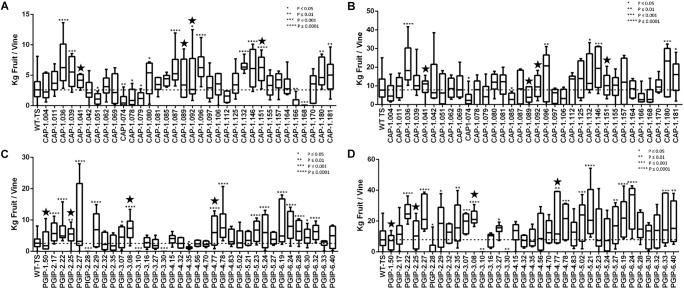
Average yield of transgenic grapevines in Field 2. **(A)** CAP-1-expressing transgenic grapevines trained with two fruiting cordons in year 6. **(B)** CAP-1-expressing transgenic grapevines trained to a single fruiting cane from the previous year’s growth in year 7. The CAP-1lines tested in Field 1 are marked with a star. **(C)** PGIP-expressing transgenic grapevines trained with two fruiting cordons in year 6. **(D)** PGIP-expressing transgenic grapevines trained to a single fruiting cane from the previous year’s growth in year 7. The PGIP lines tested in Field 1 are marked with a star.

To verify that horticultural and varietal characteristics of the transgenic rootstock lines grafted with wild type scion in Field 1 and transgenic lines on their own roots in Field 2 matched their parental TS genotype, plants were examined every year for the first 3 years after planting. This examination evaluated the first 12 descriptors from the “Primary descriptor priority list” proposed by the OIV (see Methods for a complete description). There were no differences for any descriptor between transgenic grapevines or grafted grapevines and the parental genotype TS. Grape yield was evaluated for individual vines planted in Field 2. Vines were pruned leaving one fruiting cane as done in commercial vineyards and yield was measured for all CAP and PGIP lines in year six (Figure 1A,C, respectively) and year seven (Figure 1B,D). Most CAP-expressing vines exhibited yields similar to wild type controls, but 32 and 17% produced more than controls in years six and seven, respectively, while only 17 and 7% produced less. Among the PGIP lines, 46% yielded significantly more fruit than the control both years, with 11 and 9% yielding less in years six and seven, respectively.

### Tolerance to Pierce’s Disease

**FIGURE 2:**
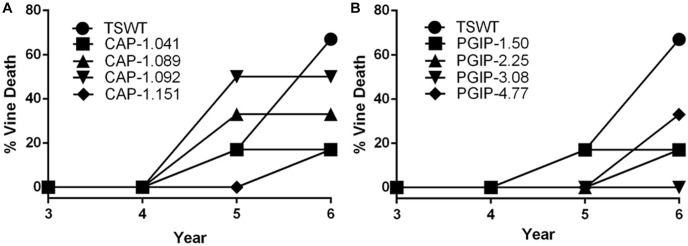
Transgraft protection against grapevine mortality after three yearly infections with *Xylella fastidiosa*. Percentage mortality for, **(A)** four CAP-I-expressing rootstocks, **(B)** four PGIP-expressing rootstocks. Mortality was calculated as a ratio of living/dead plants (*N* = 12).

**FIGURE 3:**
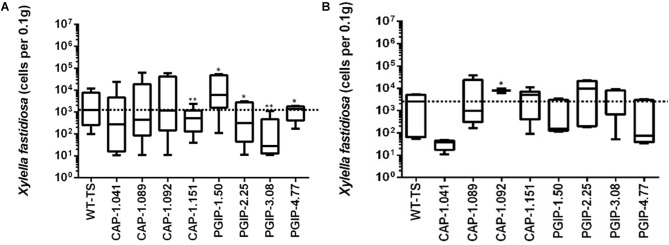
Measurement of bacterial titers at a point 10 cm above the point of infection, which was a meter above the graft union with the transgenic rootstock, in year 5. **(A)** Titer measured at 2 months, 10 cm above the point of infection and **(B)** measured at one year at the point of infection.

**FIGURE 4:**
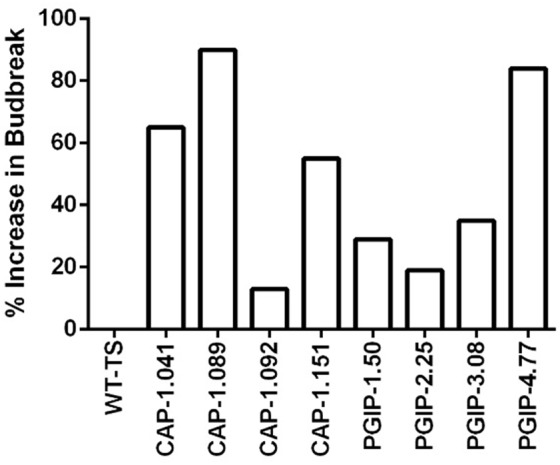
Vine health in year 5. Vine health was measured in the spring by counting the number of buds that expanded above the point of infection, as a percentage of that on control vines. Increase in bud break was calculated by first taking the average of the ratio of buds that successfully broke per cane by total buds, then setting the average of WT as the baseline, and calculating what percentage above baseline each transgenic line was. Number of plants for each line were as follows: WT-TS: 27, CAP1.041: 22, CAP1.089: 20, CAP1.092: 19, CAP1.151: 31, PGIP-1.50: 27, PGIP-2.25: 24, PGIP-3.08: 29, PGIP-4.77: 25.

The eight lines planted in Field 1 were challenged by inoculating individual shoots growing on the cordons of the wild type TS scion grafted to the transgenic rootstock. Inoculation was initiated in year 2, and each transgenic rootstock received three yearly inoculations with virulent *Xf*. The mortality in the control wild type TS grafted vines was intense: >75% of vines died by year 6 and all 12 vines by year 7, when the trial was concluded (Figure 2A). Lines CAP-1.151 and CAP-1.041 showed the greatest resistance among the CAP-1-expressing lines, with only 20% mortality at year 6. CAP-1.089 and CAP-1.092 showed moderate resistance, losing 30 and 45% of the vines by year 6, respectively (Figure 2A,B). PGIP lines showed strong resistance: line PGIP-3.08 lost no vines, PGIP-2.25 and PGIP-1.50 lost <20%, and PGIP-4.77 lost 30% by year 6 (Figure 2B). The bacterial titer was lower in the transgenic vines except in PGIP-1.50, which had a titer comparable to the control (Figure 3A). The bacterial titer was also measured at the point of inoculation 1 year after the last infection and CAP-1.041, PGIP-4.77 and PGIP-1.50 had a much lower titer than the other lines. The remaining lines had pathogen titers comparable to the control (Figure 3B). In early spring of year 5, vine health was evaluated by measuring the number of buds that successfully broke above the point of inoculation after winter dormancy as a percentage of the control. The control vines had very few buds that grew successfully: most failed to open or died afterward. Lines CAP-1.089 and PGIP-4.77 had >80% successful bud break, CAP-1.041 and CAP-1.151 had 55 to 60%, and CAP-1.092 had ∼10%, while the remaining PGIP lines had 20–30%, with PGIP-2.25 performing worst (Figure 4 and Supplementary Figure 2).

### Secretion of PGIP Into the Xylem

**FIGURE 5:**
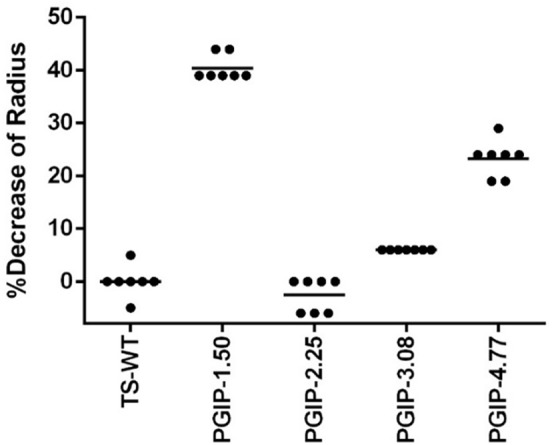
Quantification of PGIP protein secreted into the xylem during radial diffusion analysis of xylem sap extracted from transgenic PGIP-expressing grapevines in Field 2.

Since the secretion sequences were altered in the different PGIP-expressing binary vectors, we evaluated the efficiency of the various signal peptides by measuring the amount of PGIP secreted into xylem fluid. Free-flowing xylem sap was collected in early spring of year 7 from each of the vines growing in Field 2 and the percentage inhibition of PG activity isolated from *B. cinerea* was measured using a radial diffusion assay (Taylor and Secor, 1988). PGIP-1.50, which uses the endogenous native signal peptide, had the most PG activity inhibition at ∼40% (Figure 5 and Supplementary Figure 3). PGIP-2, which lacks a signal peptide, had almost no inhibition and was indistinguishable from the negative control. PGIP-3 lines also had minimal PGIP export to the xylem. However, PGIP-4 was secreted much more than PGIP-3 but less than PGIP-1. We also tested two additional PGIP lines not evaluated in Field 1, PGIP-6, which used the RAmy3D signal peptide, inhibited almost as well as PGIP-1.50 (Supplementary Figure 3). The PGIP-5 lines, created with the signal peptide from XSP, secreted PGIP slightly better than PGIP lines with no signal peptide (Supplementary Figure 3).

## Discussion

The field test described here validates, for the first time, resistance to a major aboveground plant disease (PD) provided by trans-graft protection of a susceptible scion by a transgenic rootstock. The trans-graft protection was demonstrated using two distinct strategies: expression of a CAP-1 or PGIP, which are secreted into the xylem and thus move past the graft union (Agüero et al., 2005; Dandekar et al., 2012). These two proteins mediate trans-graft resistance by disrupting the interaction between two surfaces at the interface of the host-microbe interaction. These surfaces are the lipopolysaccharide (LPS) surface of *Xf*, the causative agent of PD, and the pectin component present in the middle lamella and primary plant cell walls, that provides barrier function to sustain plant innate immunity. The evaluations in Field 2 indicate no alteration in morphology or horticultural characteristics.

### Disruption of *X. fastidiosa* LPS Disrupts PD Development

CAP-1 lines showed reduced vine mortality due to *Xf* infections than the wild type. The CAP-1 chimeric protein has two components: the *N*-terminal is a neutrophil elastase (NE) and the C-terminal is cecropin B (CecB). This chimera digests MopB, a superabundant outer membrane porin embedded in the LPS of *Xf* (Dandekar et al., 2012). MopB is very similar in structure and function to the *E. coli* OmpA protein, for which NE demonstrates a similar activity. OmpA mutants of *E. coli*, however, are not susceptible to killing by NE (Belaaouaj et al., 2000). These ompA mutants of *E. coli* are also more sensitive to oxygen free radicals and to antimicrobial peptides (Fu et al., 2003). *Xf* treated with NE and CecB in combination is more sensitive than *Xf* cells treated individually with NE or CecB (Dandekar et al., 2012). Therefore, enzymatic breakdown of MopB by NE could destabilize *Xf* LPS, making it more sensitive to the environment and the host plant immune response. MopB-deficient mutants of the Temecula-1 strain of *Xf* showed reduced virulence, twitching motility and biofilm formation (Chen et al., 2017). Outer membrane porins like MopB and OmpA play a key role in energy homeostasis by regulating the ionic flow though the LPS, which in turn regulates many bacterial functions, including cell aggregation, biofilm formation, surface attachment, twitching motility, and virulence: all features observed in *mop*B mutants of *Xf* (Chen et al., 2017). Modification of LPS properties to delay or dampen host immune responses is a virulence strategy of *Xf* (Rapicavoli et al., 2018). Enabling the host to modify the pathogen’s LPS can counter this strategy and possibly increase early responses and prevent high pathogen titers from being reached. Secretion of CAP-1 into the xylem lumen likely prevents MopB from functioning in infecting *Xf* cells, disrupting energy homeostasis of the LPS and enhancing clearance by the plant immune response. Consequently, all infected vines expressing CAP-1 were in good health in spite of the infection and showed a high frequency of spring bud break (Figure 4). Importantly, expressing CAP-1 and its secretion into the xylem does not affect grapevine productivity (Figure 1A,B). This data set evaluated 34 different CAP-1-expressing lines and indicates a low likelihood of off-target effects of CAP-1 expression on grapevine productivity.

### Preventing Pectin Disruption Provides Resistance to PD

There was very little vine mortality in all four PGIP-expressing lines. Transgenic lines PGIP-1 and PGIP-4 had both lower *Xf* titers and increased PGIP activity. PGIP-4 also displayed more bud break. Degradation of pectin-containing biopolymers and gels is important for disease development (Sun et al., 2011, 2013). *Xf* expresses a pectinase encoded by the *pglA* gene and disrupting this gene makes *Xf* avirulent to grapevines (Roper et al., 2007). *Xf* also has two fucosidases that potentially can degrade *N*-glycans decorating the pectin fibrils (Assis et al., 2017), which curiously are only present in plant-associated members of the Xanthomonadaceae family. This corroborates that pectin degradation is a virulence strategy for *Xf* and a sensitivity factor promoting disease development in grapevines. A potential key target for PglA activity is the intervessel pit membrane. It is composed of pectin and forms a physical barrier separating individual xylem elements that is likely breached to support growth and spread of *Xf* (Roper et al., 2007). Further support for this notion comes from the compositional differences in pectins composed of homogalacturonans (HGs) and xyloglucanans (XGs) that make up intervessel pit membranes of PD-sensitive vs. -resistant cultivars (Sun et al., 2011). However, three complimentary methods using bacteria-sized particles showed that intervessel pit membranes were not a factor and that unimpeded movement of these particles occurred over long distances, through stems and petioles right to the leaf edge in both a PD-sensitive and -resistant grapevine (Thorne et al., 2006). The pit membrane on the lateral wall of xylem vessels adjacent to the xylem parenchyma may be an essential feature for disease development. Masses of bacteria have been found associated to these sites and are associated with formation of tyloses and secretion of pectin-rich gels that occlude xylem vessels, restricting the flow of water and leading to vine death (Sun et al., 2013). Tylose formation is not restricted to PD, but occurs in response to stresses and is mediated by ethylene (Sun et al., 2006, 2007). It is likely that these pathological processes are limited in PGIP-expressing grapevine lines, dramatically lowering incidence of vine death (Figure 2B) and promoting strong spring bud break (Figure 4), indicating unimpeded xylem flow. Interestingly, PGIP-2.25, which has no signal peptide sequence, and PGIP-3.08, which contains the NT-signal, showed little PGIP activity in the xylem sap, but also decreased plant mortality. While the secretion sequence may be significant, PGIP without a secretion signal appears to work indirectly to provide resistance to PD. PGIP are leucine-rich repeat proteins (LRR), much like pattern-recognition immune receptors and R-genes, and therefore stimulate the innate defense response (Federici et al., 2006). Structural analysis of the LRR domain of PGIP revealed that it is very adaptable and evolves quickly in response to changes in ligand structure (Casasoli et al., 2009). This aspect may explain how the recombinant protein expressed in line PGIP-2.25, although missing a signal peptide, provides resistance to *Xf* infection (Figure 2B). In this line, PGIP is not secreted, but accumulates in the cytoplasm, where it activates the plant innate defense response by virtue of its LRR interactions as described (Federici et al., 2006). This upregulation of innate immunity would be an additive effect of the LRR domain in addition to blocking pectin breakdown and could account for the significant yield increases in 16 of the 35 PGIP-expressing lines evaluated (Figure 1C,D).

## Conclusion

This work shows the potential of trans-graft protection as a tool to increase disease resistance without genetic modification of the scion. Since most specialty crops rely on grafting systems, this approach can be extended to other species for which preservation of the scion genotype is desired or where genetically modified scions are prohibited by regulations or rejected by consumers. We demonstrated that the interaction between host and pathogen can be disturbed on different levels, which stimulates novel research to better understand and reduce pathogen fitness. A combination of approaches may provide even greater resistance, as one might expect from a line expressing both CAP and PGIP. Efforts to stack resistance are underway in our group. Our experimental design demonstrates that trans-graft protection is effective under field conditions. This can be adopted by integrated pest management efforts to offer growers an alternative to reduce disease incidence without heavy dependence on insecticides to control the insect vectors. This not only reduces production costs and increases grower profitability, but also protects consumers and the environment from toxic pesticides.

## Author Contributions

AD designed the study. CA and SU built the vectors. AJ, AI, HG, DD, and RJ performed the research. AD, AJ, AI, and PZ analyzed and interpreted the data. AD wrote the first draft with contributions from PZ, AJ, AI, CA, and SU. All authors read and approved the final manuscript.

## Conflict of Interest Statement

The authors declare that the research was conducted in the absence of any commercial or financial relationships that could be construed as a potential conflict of interest.

## Footnotes

1 ftp://occams.dfci.harvard.edu/pub/bio/tgi/data/Vitis_vinifera/
