# Supplementary material for: Trans-Graft Protection Against Pierce’s Disease Mediated by Transgenic Grapevine Rootstocks
Source: Front Plant Sci. 2019 Feb 6;10:84. doi: 10.3389/fpls.2019.00084 (PMC6372540; doi:10.3389/fpls.2019.00084)

Supplementary Materials:

Supplementary Table 1: Field map of genotypes planted in Field 1.

Vine	1	2	3	4	5	6	7	8	9	10	11	12	13	14	15	16	17	18	19	20	21	22	23	24	25
**Row 9**	CAP-1.041	CAP-1.041	PGIP-1.50	PGIP-1.50	PGIP-1.50	CAP-1.092	CAP-1.092	CAP-1.092	CAP-1.089	CAP-1.089	CAP-1.089	TS-WT	TS-WT	TS-WT	PGIP-4.77	PGIP-4.77	PGIP-4.77	CAP-1.151	CAP-1.151	CAP-1.151	TS-WT	TS-WT	TS-WT	TS-WT	
**Row 8**	PGIP-3.08	PGIP-3.08	PGIP-3.08	PGIP-2.25	PGIP-2.25	PGIP-2.25	CAP-1.041	CAP-1.041	CAP-1.041	PGIP-1.50	PGIP-1.50	PGIP-1.50	CAP-1.089	CAP-1.089	CAP-1.089	TS-WT	TS-WT	TS-WT	PGIP-3.08	PGIP-3.08	PGIP-3.08	PGIP-2.25	PGIP-2.25	PGIP-2.25	CAP-1.041
**Row 7**	PGIP-2.25	CAP-1.092	CAP-1.092	CAP-1.092	PGIP-1.50	PGIP-1.50	PGIP-1.50	CAP-1.089	CAP-1.089	CAP-1.089	TS-WT	TS-WT	TS-WT	PGIP-4.77	PGIP-4.77	PGIP-4.77	CAP-1.092	CAP-1.092	CAP-1.092	CAP-1.151	CAP-1.151	CAP-1.151	PGIP-4.77	PGIP-4.77	PGIP-4.77
**Row 6**	PGIP-1.50	PGIP-1.50	CAP-1.151	CAP-1.151	CAP-1.151	TS-WT	TS-WT	TS-WT	CAP-1.092	CAP-1.092	CAP-1.092	CAP-1.089	CAP-1.089	CAP-1.089	CAP-1.151	CAP-1.151	CAP-1.151	PGIP-3.08	PGIP-3.08	PGIP-3.08	CAP-1.041	CAP-1.041	CAP-1.041	PGIP-2.25	PGIP-2.25
**Row 5**													PGIP-3.08	PGIP-3.08	PGIP-3.08	CAP-1.041	CAP-1.041	CAP-1.041	PGIP-2.25	PGIP-2.25	PGIP-2.25	PGIP-4.77	PGIP-4.77	PGIP-4.77	PGIP-1.50

Supplementary Table 2: Transgenic grapevine genotypes planted in Field 2.

No	Line Designation	Binary Vector	Transgene	Event No.	No of Plants
1	CAP-1.004	pDU04.6105	HNE-CecB	0999040-04	6
2	CAP-1.011	pDU04.6105	HNE-CecB	0999040-11	6
3	CAP-1.036	pDU04.6105	HNE-CecB	0999040-36	6
4	CAP-1.039	pDU04.6105	HNE-CecB	0999040-39	6
5	CAP-1.041	pDU04.6105	HNE-CecB	0999040-41	6
6	CAP-1.042	pDU04.6105	HNE-CecB	0999040-42	6
7	CAP-1.051	pDU04.6105	HNE-CecB	0999040-51	6
8	CAP-1.062	pDU04.6105	HNE-CecB	0999040-62	6
9	CAP-1.069	pDU04.6105	HNE-CecB	0999040-69	6
10	CAP-1.074	pDU04.6105	HNE-CecB	0999040-74	6
11	CAP-1.078	pDU04.6105	HNE-CecB	0999040-78	6
12	CAP-1.079	pDU04.6105	HNE-CecB	0999040-79	6
13	CAP-1.080	pDU04.6105	HNE-CecB	0999040-80	6
14	CAP-1.081	pDU04.6105	HNE-CecB	0999040-81	6

15	CAP-1.085	pDU04.6105	HNE-CecB	0999040-85	6
16	CAP-1.087	pDU04.6105	HNE-CecB	0999040-87	6
17	CAP-1.089	pDU04.6105	HNE-CecB	0999040-89	6
18	CAP-1.092	pDU04.6105	HNE-CecB	0999040-92	6
19	CAP-1.096	pDU04.6105	HNE-CecB	0999040-96	6
20	CAP-1.097	pDU04.6105	HNE-CecB	0999040-97	6
21	CAP-1.106	pDU04.6105	HNE-CecB	0999040-106	6
22	CAP-1.112	pDU04.6105	HNE-CecB	0999040-112	6
23	CAP-1.125	pDU04.6105	HNE-CecB	0999041-125	6
24	CAP-1.132	pDU04.6105	HNE-CecB	0999041-132	6
25	CAP-1.146	pDU04.6105	HNE-CecB	0999041-146	6
26	CAP-1.151	pDU04.6105	HNE-CecB	0999041-151	6
27	CAP-1.155	pDU04.6105	HNE-CecB	0999041-155	6
28	CAP-1.157	pDU04.6105	HNE-CecB	0999041-157	6
29	CAP-1.164	pDU04.6105	HNE-CecB	0999041-164	6
30	CAP-1.166	pDU04.6105	HNE-CecB	0999041-166	6
31	CAP-1.168	pDU04.6105	HNE-CecB	0999041-168	6
32	CAP-1.179	pDU04.6105	HNE-CecB	0999041-179	6
33	CAP-1.180	pDU04.6105	HNE-CecB	0999041-180	6
34	CAP-1.181	pDU04.6105	HNE-CecB	0999041-181	6
35	CAP-1.007	pDA05.0525	pHNE-CecB	099944-007	6
36	CAP-1.002	pDA05.0525	pHNE-CecB	099946-002	6
37	CAP-1.014	pDA05.0525	pHNE-CecB	099946-014	6
38	CAP-1.018	pDA05.0525	pHNE-CecB	099946-018	6
39	CAP-1.019	pDA05.0525	pHNE-CecB	099946-019	6
40	CAP-1.016	pDA05.0525	pHNE-CecB	061073-016	6
41	CAP-1.028	pDA05.0525	pHNE-CecB	061073-028	6
42	CAP-1.036	pDA05.0525	pHNE-CecB	061073-036	6
43	PGIP-1.50	pDU94.0928	pearPGIP	TS-50	6
44	PGIP-2.17	pDU05.1002	mPGIP	061131-17	6
45	PGIP-2.22	pDU05.1002	mPGIP	061131-22	6
46	PGIP-2.25	pDU05.1002	mPGIP	061131-25	6
47	PGIP-2.27	pDU05.1002	mPGIP	061131-27	6
48	PGIP-2.28	pDU05.1002	mPGIP	061131-28	6
49	PGIP-2.29	pDU05.1002	mPGIP	061131-29	6
50	PGIP-2.32	pDU05.1002	mPGIP	061131-32	6
51	PGIP-2.35	pDU05.1002	mPGIP	061131-35	6
52	PGIP-3.07	pDU05.1910	ntPGIP	061034-007	6
53	PGIP-3.08	pDU05.1910	ntPGIP	061052-008	6
54	PGIP-3.10	pDU05.1910	ntPGIP	061034-010	6
55	PGIP-3.16	pDU05.1910	ntPGIP	061052-016	6
56	PGIP-3.27	pDU05.1910	ntPGIP	061036-27	6
57	PGIP-3.30	pDU05.1910	ntPGIP	061036-30	6
58	PGIP-4.15	pDU06.0201	chiPGIP	061045-015	6
59	PGIP-4.32	pDU06.0201	chiPGIP	061045-032	6

60	PGIP-4.35	pDU06.0201	chiPGIP	061045-035	6
61	PGIP-4.56	pDU06.0201	chiPGIP	061045-056	6
62	PGIP-4.70	pDU06.0201	chiPGIP	061045-070	6
63	PGIP-4.77	pDU06.0201	chiPGIP	061045-077	6
64	PGIP-4.78	pDU06.0201	chiPGIP	061045-078	6
65	PGIP-4.83	pDU06.0201	chiPGIP	061045-083	6
66	PGIP-5.02	pDA05.XSP	xspPGIP	061133-02	6
67	PGIP-5.21	pDA05.XSP	xspPGIP	061133-21	6
68	PGIP-5.23	pDA05.XSP	xspPGIP	061133-23	6
69	PGIP-5.24	pDA05.XSP	xspPGIP	061133-24	6
70	PGIP-5.27	pDA05.XSP	xspPGIP	061133-27	6
71	PGIP-6.19	pDU05.0401	RamyPGIP	061132-19	6
72	PGIP-6.24	pDU05.0401	RamyPGIP	061132-24	6
73	PGIP-6.28	pDU05.0401	RamyPGIP	061132-28	5
74	PGIP-6.30	pDU05.0401	RamyPGIP	061132-30	6
75	PGIP-6.32	pDU05.0401	RamyPGIP	061132-32	6
76	PGIP-6.33	pDU05.0401	RamyPGIP	061173-33	6
77	PGIP-6.40	pDU05.0401	RamyPGIP	061173-40	4
78	WT-TS				140

Supplementary Table 3: Field map of genotypes planted in Field 2.

**Row 1**	**Row 2**	**Row 3**	**Row 4**	**Row 5**	**Row 6**	**Row 7**	**Row 8**	**Row 9**	**Row 10**
TS-WT	TS-WT	CAP-1.007	CAP-1.080	PGIP-2.22	PGIP-2.28	CAP-1.036	CAP-1.125	PGIP-4.35	PGIP-5.02
TS-WT	TS-WT	CAP-1.007	CAP-1.080	PGIP-2.22	PGIP-2.28	CAP-1.036	CAP-1.125	PGIP-4.35	PGIP-5.02
TS-WT	TS-WT	CAP-1.007	CAP-1.080	PGIP-2.22	PGIP-2.28	CAP-1.036	CAP-1.125	PGIP-4.35	PGIP-5.02
TS-WT	TS-WT	CAP-1.002	CAP-1.081	CAP-1.180	PGIP-2.29	CAP-1.039	CAP-1.132	PGIP-4.56	PGIP-5.27
TS-WT	TS-WT	CAP-1.002	CAP-1.081	CAP-1.180	PGIP-2.29	CAP-1.039	CAP-1.132	PGIP-4.56	PGIP-5.27
TS-WT	TS-WT	CAP-1.002	CAP-1.081	CAP-1.180	PGIP-2.29	CAP-1.039	CAP-1.132	PGIP-4.56	PGIP-5.27
TS-WT	TS-WT	CAP-1.014	CAP-1.085	CAP-1.181	PGIP-2.32	CAP-1.041	CAP-1.146	PGIP-4.70	PGIP-6.19
TS-WT	TS-WT	CAP-1.014	CAP-1.085	CAP-1.181	PGIP-2.32	CAP-1.041	CAP-1.146	PGIP-4.70	PGIP-6.19
TS-WT	TS-WT	CAP-1.014	CAP-1.085	CAP-1.181	PGIP-2.32	CAP-1.041	CAP-1.146	PGIP-4.70	PGIP-6.19
TS-WT	TS-WT	CAP-1.018	CAP-1.087	PGIP-3.07	PGIP-2.35	CAP-1.042	CAP-1.151	PGIP-4.77	PGIP-6.24
TS-WT	TS-WT	CAP-1.018	CAP-1.087	PGIP-3.07	PGIP-2.35	CAP-1.042	CAP-1.151	PGIP-4.77	PGIP-6.24
TS-WT	TS-WT	CAP-1.018	CAP-1.087	PGIP-3.07	PGIP-2.35	CAP-1.042	CAP-1.151	PGIP-4.77	PGIP-6.24
TS-WT	TS-WT	CAP-1.019	CAP-1.089	PGIP-3.10	PGIP-6.30	CAP-1.051	CAP-1.155	PGIP-4.78	PGIP-6.28
TS-WT	TS-WT	CAP-1.019	CAP-1.089	PGIP-3.10	PGIP-6.30	CAP-1.051	CAP-1.155	PGIP-4.78	PGIP-6.28
TS-WT	TS-WT	CAP-1.019	CAP-1.089	PGIP-3.10	PGIP-6.30	CAP-1.051	CAP-1.155	PGIP-4.78	PGIP-6.28
TS-WT	TS-WT	CAP-1.016	CAP-1.092	PGIP-3.27	PGIP-6.32	CAP-1.062	CAP-1.157	PGIP-4.83	PGIP-6.33
TS-WT	TS-WT	CAP-1.016	CAP-1.092	PGIP-3.27	PGIP-6.32	CAP-1.062	CAP-1.157	PGIP-4.83	PGIP-6.33
TS-WT	TS-WT	CAP-1.016	CAP-1.092	PGIP-3.27	PGIP-6.32	CAP-1.062	CAP-1.157	PGIP-4.83	PGIP-6.33
TS-WT	TS-WT	CAP-1.028	CAP-1.096	PGIP-3.30	PGIP-5.21	CAP-1.069	CAP-1.164	PGIP-2.17	

TS-WT	TS-WT	CAP-1.028	CAP-1.096	PGIP-3.30	PGIP-5.21	CAP-1.069	CAP-1.164	PGIP-2.17	PGIP-6.40
TS-WT	TS-WT	CAP-1.028	CAP-1.096	PGIP-3.30	PGIP-5.21	CAP-1.069	CAP-1.164	PGIP-2.17	
TS-WT	TS-WT	CAP-1.036	CAP-1.097	PGIP-3.08	PGIP-5.23	CAP-1.074	CAP-1.166	PGIP-2.22	
TS-WT	TS-WT	CAP-1.036	CAP-1.097	PGIP-3.08	PGIP-5.23	CAP-1.074	CAP-1.166	PGIP-2.22	
TS-WT	TS-WT	CAP-1.036	CAP-1.097	PGIP-3.08	PGIP-5.23	CAP-1.074	CAP-1.166	PGIP-2.22	
TS-WT	TS-WT	CAP-1.004	CAP-1.106	PGIP-3.16	PGIP-5.24	CAP-1.078	CAP-1.168	PGIP-2.25	
TS-WT	TS-WT	CAP-1.004	CAP-1.106	PGIP-3.16	PGIP-5.24	CAP-1.078	CAP-1.168	PGIP-2.25	
TS-WT	TS-WT	CAP-1.004	CAP-1.106	PGIP-3.16	PGIP-5.24	CAP-1.078	CAP-1.168	PGIP-2.25	
TS-WT	TS-WT	CAP-1.011	CAP-1.112	PGIP-4.15	PGIP-1.50	CAP-1.079	CAP-1.179	PGIP-2.27	
TS-WT	TS-WT	CAP-1.011	CAP-1.112	PGIP-4.15	PGIP-1.50	CAP-1.079	CAP-1.179	PGIP-2.27	
TS-WT	TS-WT	CAP-1.011	CAP-1.112	PGIP-4.15	PGIP-1.50	CAP-1.079	CAP-1.179	PGIP-2.27	
TS-WT	TS-WT	CAP-1.036	CAP-1.125	PGIP-4.32	CAP-1.007	CAP-1.080	CAP-1.180	PGIP-2.28	PGIP-5.02
TS-WT	TS-WT	CAP-1.036	CAP-1.125	PGIP-4.32	CAP-1.007	CAP-1.080	CAP-1.180	PGIP-2.28	PGIP-5.02
TS-WT	TS-WT	CAP-1.036	CAP-1.125	PGIP-4.32	CAP-1.007	CAP-1.080	CAP-1.180	PGIP-2.28	PGIP-5.02
TS-WT	TS-WT	CAP-1.039	CAP-1.132	PGIP-4.35	CAP-1.002	CAP-1.081	CAP-1.181	PGIP-2.29	PGIP-5.27
TS-WT	TS-WT	CAP-1.039	CAP-1.132	PGIP-4.35	CAP-1.002	CAP-1.081	CAP-1.181	PGIP-2.29	PGIP-5.27
TS-WT	TS-WT	CAP-1.039	CAP-1.132	PGIP-4.35	CAP-1.002	CAP-1.081	CAP-1.181	PGIP-2.29	PGIP-5.27
TS-WT	TS-WT	CAP-1.041	CAP-1.146	PGIP-4.56	CAP-1.014	CAP-1.085	PGIP-3.07	PGIP-2.32	PGIP-6.19
TS-WT	TS-WT	CAP-1.041	CAP-1.146	PGIP-4.56	CAP-1.014	CAP-1.085	PGIP-3.07	PGIP-2.32	PGIP-6.19
TS-WT	TS-WT	CAP-1.041	CAP-1.146	PGIP-4.56	CAP-1.014	CAP-1.085	PGIP-3.07	PGIP-2.32	PGIP-6.19
TS-WT	TS-WT	CAP-1.042	CAP-1.151	PGIP-4.70	CAP-1.018	CAP-1.087	PGIP-3.10	PGIP-2.35	PGIP-6.24
TS-WT	TS-WT	CAP-1.042	CAP-1.151	PGIP-4.70	CAP-1.018	CAP-1.087	PGIP-3.10	PGIP-2.35	
TS-WT	TS-WT	CAP-1.042	CAP-1.151	PGIP-4.70	CAP-1.018	CAP-1.087	PGIP-3.10	PGIP-2.35	PGIP-6.24
TS-WT	TS-WT	CAP-1.051	CAP-1.155	PGIP-4.77	CAP-1.019	CAP-1.089	PGIP-3.27	PGIP-6.30	PGIP-6.28
TS-WT	TS-WT	CAP-1.051	CAP-1.155	PGIP-4.77	CAP-1.019	CAP-1.089	PGIP-3.27	PGIP-6.30	PGIP-6.28
TS-WT	TS-WT	CAP-1.051	CAP-1.155	PGIP-4.77	CAP-1.019	CAP-1.089	PGIP-3.27	PGIP-6.30	PGIP-6.28
TS-WT	TS-WT	CAP-1.062	CAP-1.157	PGIP-4.78	CAP-1.016	CAP-1.092	PGIP-3.30	PGIP-6.32	PGIP-6.33
TS-WT	TS-WT	CAP-1.062	CAP-1.157	PGIP-4.78	CAP-1.016	CAP-1.092	PGIP-3.30	PGIP-6.32	PGIP-6.33
TS-WT	TS-WT	CAP-1.062	CAP-1.157	PGIP-4.78	CAP-1.016	CAP-1.092	PGIP-3.30	PGIP-6.32	PGIP-6.33
TS-WT	TS-WT	CAP-1.069	CAP-1.164	PGIP-4.83	CAP-1.028	CAP-1.096	PGIP-3.08	PGIP-5.21	PGIP-6.40
TS-WT	TS-WT	CAP-1.069	CAP-1.164	PGIP-4.83	CAP-1.028	CAP-1.096	PGIP-3.08	PGIP-5.21	PGIP-6.40
TS-WT	TS-WT	CAP-1.069	CAP-1.164	PGIP-4.83	CAP-1.028	CAP-1.096	PGIP-3.08	PGIP-5.21	PGIP-6.40
TS-WT	TS-WT	CAP-1.074	CAP-1.166	PGIP-2.17	CAP-1.036	CAP-1.097	PGIP-3.16	PGIP-5.23	
TS-WT	TS-WT	CAP-1.074	CAP-1.166	PGIP-2.17	CAP-1.036	CAP-1.097	PGIP-3.16	PGIP-5.23	
TS-WT	TS-WT	CAP-1.074	CAP-1.166	PGIP-2.17	CAP-1.036	CAP-1.097	PGIP-3.16	PGIP-5.23	
TS-WT	TS-WT	CAP-1.078	CAP-1.168	PGIP-2.25	CAP-1.004	CAP-1.106	PGIP-4.15	PGIP-5.24	
TS-WT	TS-WT	CAP-1.078	CAP-1.168	PGIP-2.25	CAP-1.004	CAP-1.106	PGIP-4.15	PGIP-5.24	
TS-WT	TS-WT	CAP-1.078	CAP-1.168	PGIP-2.25	CAP-1.004	CAP-1.106	PGIP-4.15	PGIP-5.24	
TS-WT	TS-WT	CAP-1.079	CAP-1.179	PGIP-2.27	CAP-1.011	CAP-1.112	PGIP-4.32	PGIP-1.50	
TS-WT	TS-WT	CAP-1.079	CAP-1.179	PGIP-2.27	CAP-1.011	CAP-1.112	PGIP-4.32	PGIP-1.50	
TS-WT	TS-WT	CAP-1.079	CAP-1.179	PGIP-2.27	CAP-1.011	CAP-1.112	PGIP-4.32	PGIP-1.50	

TS-WT	TS-WT	TS-WT	TS-WT	TS-WT	TS-WT	TS-WT	TS-WT	TS-WT	TS-WT
TS-WT	TS-WT	TS-WT	TS-WT	TS-WT	TS-WT	TS-WT	TS-WT	TS-WT	TS-WT

Supplementary Figures:

Supplementary Fig 1: Maps of the binary vectors used to create the lines tested in this study.

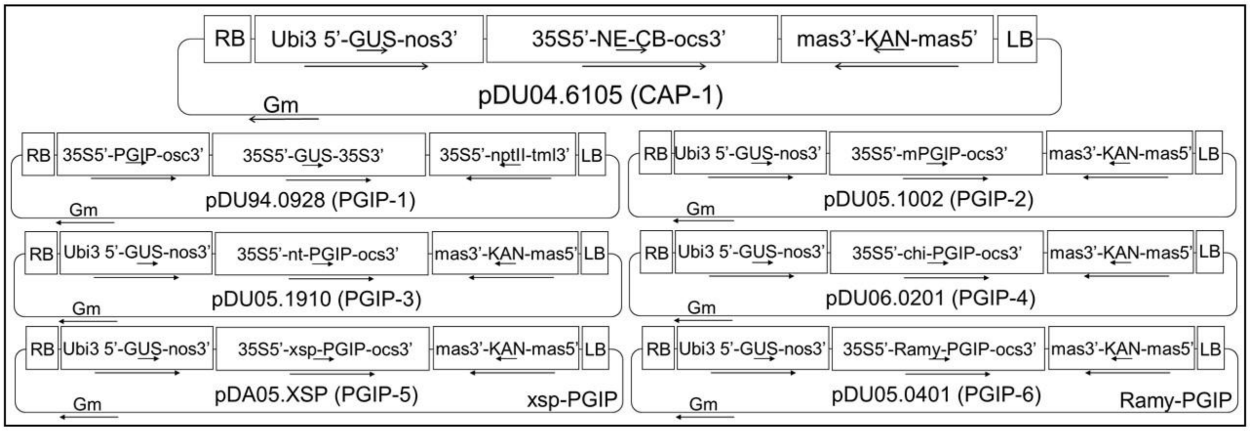

Supplementary Fig 2: Observed frequency of bud break in each infected cane in Field 1 in year four.

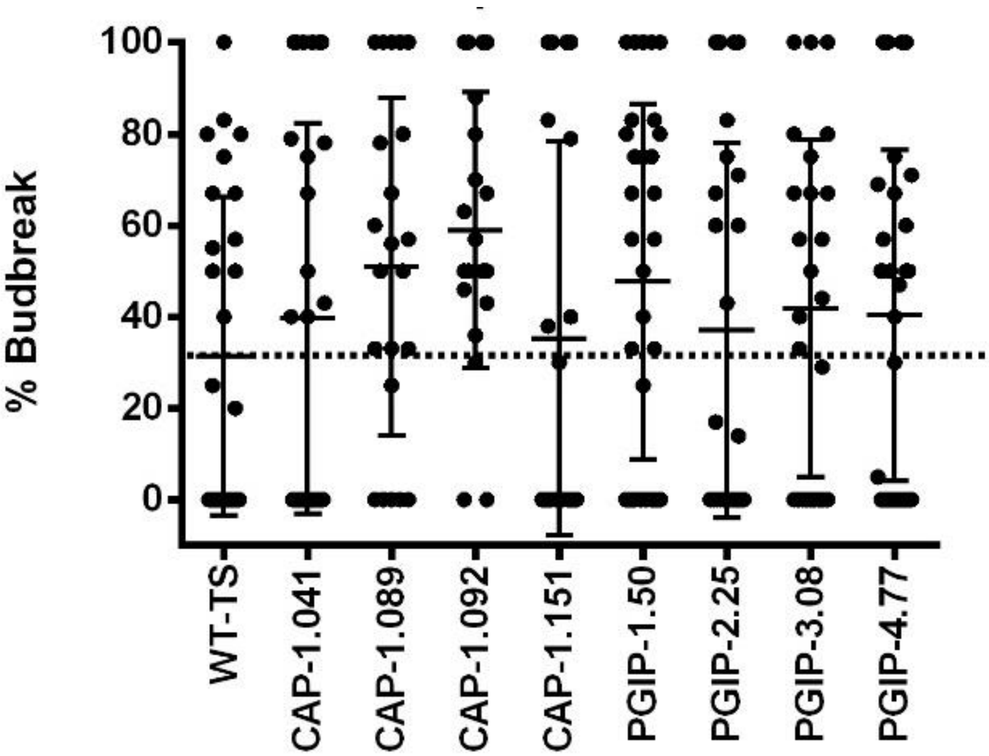

Supplementary Figure 3: Quantification of PGIP protein secreted into the xylem during radial diffusion analysis of xylem sap extracted from transgenic PGIP-expressing grapevines in Field 2.

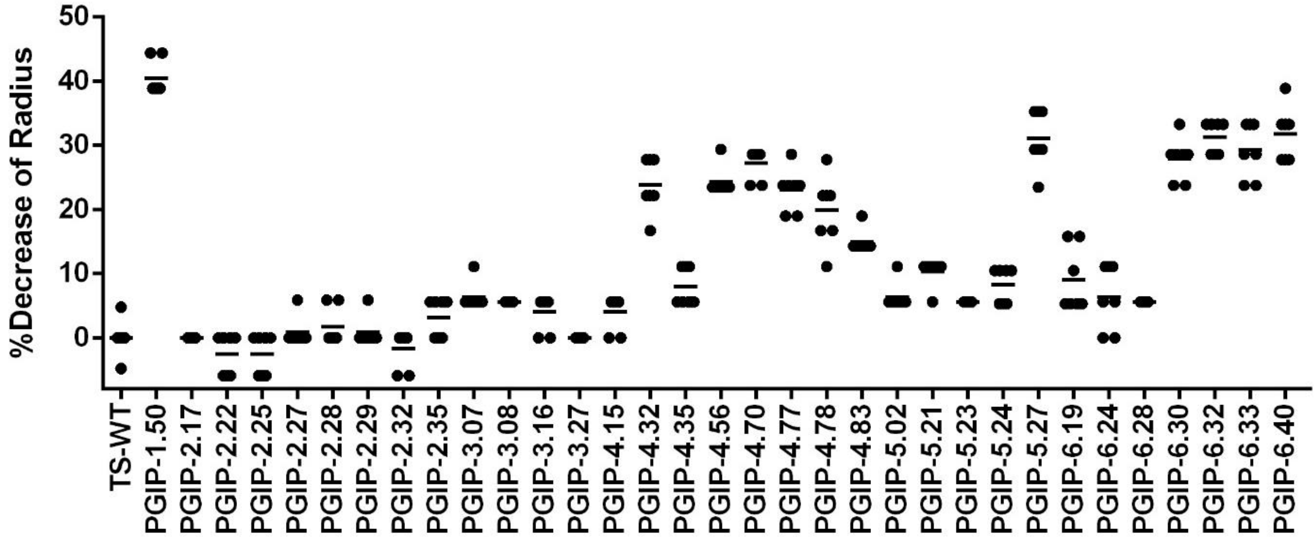

Supplement: Supplementary file 1 [file Data_Sheet_1.pdf]
